# P-620. Epidemiological Profile of Patients with Pulmonary Tuberculosis in a Tertiary Care Hospital Over 5 Years

**DOI:** 10.1093/ofid/ofaf695.833

**Published:** 2026-01-11

**Authors:** Mayra Evelyn Quiñones-Martínez, Luis Del Carpio-Orantes, René García-Toral, Verónica Montes-Martínez, José Luis Torres-Sánchez, Leopoldo Díaz-Aguilar

**Affiliations:** Hospital de Alta Especialidad de Veracruz, Veracruz, Veracruz-Llave, Mexico; Grupo de Estudio para el Diagnóstico y Tratamiento de COVID-19 en Veracruz, México, Veracruz, Veracruz-Llave, Mexico; Hospital de Alta Especialidad de Veracruz, Veracruz, Veracruz-Llave, Mexico; Hospital de Alta Especialidad de Veracruz, Veracruz, Veracruz-Llave, Mexico; Hospital de Alta Especialidad de Veracruz, Veracruz, Veracruz-Llave, Mexico; Hospital de Alta Especialidad de Veracruz, Veracruz, Veracruz-Llave, Mexico

## Abstract

**Background:**

Following the COVID-19 pandemic, the incidence and prevalence rates of tuberculosis have increased globally; therefore, it is important to determine the epidemiological profile of these patients, primarily in populations endemic for this mycobacterial infection.

**Figure:**
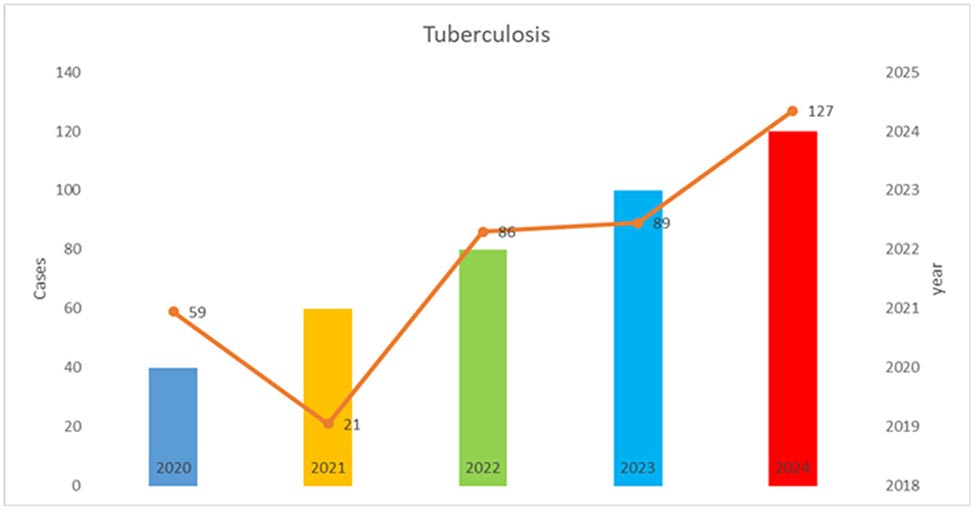
Cases of tuberculosis per year

**Figure:**
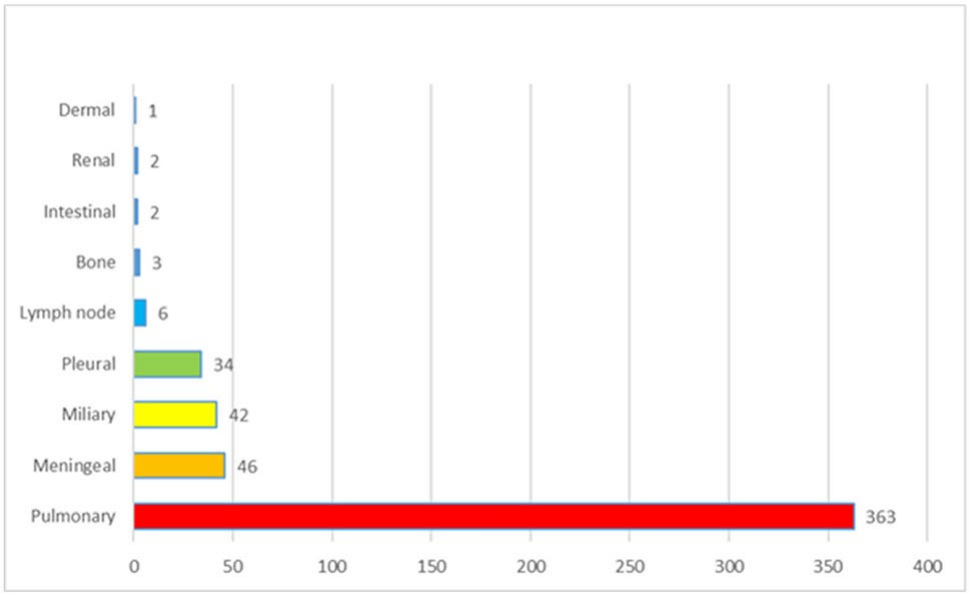
Anatomical involvement by tuberculosis

**Methods:**

Epidemiological reports of tuberculosis cases in the pediatric and adult population from 2020 to 2024 in a tertiary care hospital in Veracruz, Mexico, were analyzed.

**Results:**

847 suspected cases were reported, 506 cases were confirmed (60%). The average age was 42 years (range, 1 to 81 years). 70% of cases were male. The distribution of cases/year was: 2020: 59 cases, 2021: 21 cases, 2022: 86 cases, 2023: 89 cases, 2024: 127 cases. Pulmonary tuberculosis was reported in 72% cases, followed by meningeal tuberculosis 9%, miliary 8%, pleural 7%, lymph node 1%, bone 0.5%. New patients entering treatment 327 cases (65%), new patients with drug resistance 2%, Relapse to primary treatment 1%, Relapse to primary retreatment 1%, Readmission due to abandonment of primary treatment 3%, Readmission due to abandonment of primary retreatment 1%, Readmission due to failure of primary treatment 1%, Readmission due to failure of primary retreatment 0.7%. Patients were cured in 10%, death from tuberculosis in 3.3%, and death from other causes in 14%. Treatment failure in 0.3%, treatment abandonment in 7.3%, treatment completion in 12%, and current treatment in 24%. Drug resistance analysis included 154 cases (30%), mono-resistant in 1%, and treatment-sensitive in 29%.

**Conclusion:**

It is important to understand the epidemiological profile of tuberculosis in endemic areas in order to implement better strategies focused on the surveillance, diagnosis, and treatment of this infectious disease, and to limit treatment failure, abandonment, and resistance.

**Disclosures:**

All Authors: No reported disclosures

